# Research on roller monitoring technology based on distributed fiber optic sensing system

**DOI:** 10.1038/s41598-024-60884-z

**Published:** 2024-05-02

**Authors:** Miao Xie, Bo Li, Suning Ma, Jinnan Lu, Guilin Hu, Qingshuang Meng, Jiaxing Luo

**Affiliations:** 1 School of Mechanical Engineering, Liaoning Technical University, Fuxin, 123000 China; 2 Faculty of Electrical and Control Engineering, Liaoning Technical University, Huludao, 125105 China; 3 Xinjiang Key Laboratory of Intelligent Exploit and Control of Open-pit Mine, Changji, 831100 China

**Keywords:** Engineering, Mechanical engineering

## Abstract

As one of the key components of the belt conveyor, the roller bears the task of supporting and rolling the conveyor belt, and monitoring its condition is very important. The traditional monitoring of the conveyor roller group adopts worker inspection, which has strong subjectivity. Monitoring using sensors necessitates the use of numerous sensors, which can pose wiring challenges. The use of inspection robots for monitoring results can be discontinuous, and their performance may be limited. This study proposes a fault diagnosis method for rollers based on a distributed fiber optic sensing system. By improving the traditional Isolation Forest (IForest), a framework called Incremental Majority Voting Isolation Forest (IMV-IForest) is proposed. By analyzing the optical signal, we extracted the variation patterns of roller faults over time and space, and analyzed the abnormal score distribution between fault data and normal data. Using the dataset collected on-site, we compared and analyzed IMV-IForest with the traditional IForest and the Extended Isolation Forest (E-iForest). The results indicate that the variation of the fault of the faulty roller with time and space can be used for early prediction of roller faults; determine an anomaly score threshold of 0.6; improved IForest have faster computation time and higher accuracy. Finally, to verify the effectiveness of the proposed scheme, a 3-month experiment was conducted on a 600 m long belt conveyor in a certain mine, and on-site monitoring results were obtained. By comparing with manual detection results, it was shown that the proposed method has high recognition rate for faulty idlers, with an accuracy rate of 97.92%, and can effectively diagnose faulty idlers.

## Introduction

The belt conveyor is an essential material handling equipment widely utilized in various industrial production fields such as mines, power plants, ports, and so on. Among its crucial components, the roller plays a vital role in supporting and rolling the conveyor belt. However, due to prolonged usage and environmental factors, the support rollers are susceptible to malfunctions. Once a malfunction occurs, it can easily lead to downtime and reduced production efficiency, potentially resulting in equipment damage and even casualties. Consequently, monitoring and diagnosing the support rollers are of utmost importance^1^.

**Figure 1:**
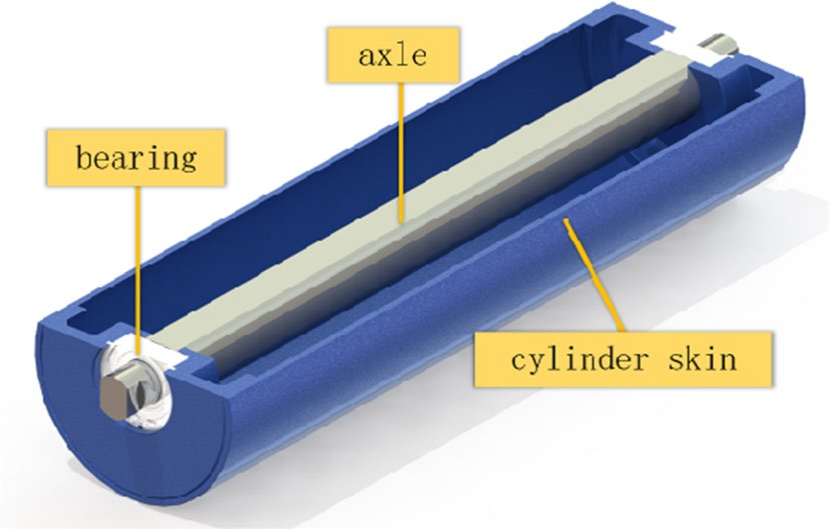
Idler structure diagram.

Idlers are mainly divided into two types: load-bearing idlers and non-load-bearing idlers. The roller primarily consists of bearings and shells, as depicted in Fig. 1. Both structures may fail, mainly due to roller skin fracture and bearing damage. Roller failure is one of the main causes of conveyor failures and also the primary cause of conveyor fires.

Therefore, monitoring the status of the rollers is crucial for ensuring smooth mining, reducing safety risks and economic losses in the mine. The traditional monitoring method for conveyor roller groups includes workers walking along the conveyor and detecting them visually and audibly. However, this detection method has subjective properties and can produce unreliable diagnostic results^2^. Some coal mines are monitored through the placement of sensors locally. However, due to rollers distributed over vast distances, many sensors need to be arranged, and wiring is also difficult. In addition, some coal mines install inspection robots to inspect belt conveyors, but the inspection robots stay near individual rollers for a short time, which may prevent timely detection of faults and limit their performance. Therefore, monitoring the rollers is a particularly challenging. In addition, it is necessary to conduct early detection of roller faults in order to arrange maintenance time reasonably and reduce the impact on the overall operating performance of the conveyor.

In terms of anomaly detection, based on whether the sample labels are involved, anomaly detection methods can be divided into supervised learning and unsupervised learning. Unsupervised learning anomaly detection methods are more widely used in anomaly detection research. Niu Gang^3^ et al. used traditional IForest to detect anomalies in the power system, but its data convergence speed was slow. Ding^4^ et al. proposed a pruning histogram IForest to improve the detection performance of the IForest algorithm, but it takes longer to compute in datasets with large amounts of data. Gao^5^ et al. proposed a k-means based IForest Algorithm, which can effectively detect local anomalies. However, due to some shortcomings of K-means, this algorithm cannot be applied to large-scale or complex datasets. Hariri^6^ et al. proposed the E-IForest algorithm, which solves the problem of insensitivity to local outliers in the IForest algorithm. However, the E-IForest algorithm has high computational overhead and insufficient accuracy and stability.

In terms of abnormal monitoring of conveyors, Yang^7^ reported the successful application of DOFS for temperature monitoring of mining conveyors in Queensland, Australia. Guo Qinghua^8^ studied the temperature fault of the roller shaft of a belt conveyor using distributed fiber optic temperature measurement technology based on the principle of heat conduction. Temperature monitoring can effectively prevent fires from occurring, but it cannot detect early faults. Compared to temperature, sound signals usually have a smaller signal-to-noise ratio and can provide better features to detect faults. Hao Hongtao^9^ et al. used LabVIEW software as the development platform to study a fault diagnosis method for rollers based on sound signals, and verified the effectiveness of the fault diagnosis method for rollers based on sound signals through experiments. Zhang Gaoxiang^10^ designed a roller fault detection system and studied the main faults of the rollers, such as the outer ring fault of the roller bearing, the inner ring fault of the roller bearing, and the rolling element fault of the roller bearing. Wu Guoping^11^ collected audio signals from the rollers running along the belt conveyor using a pickup, and proposed a coal conveyor roller fault detection method based on fusion signal and multi input one-dimensional convolutional neural network. Wijaya^12^ studied the application of artificial neural networks to classify conveyor faults of different levels under different working conditions. This study is limited to experimental environments and cannot represent on-site conditions.

This article proposes a fault diagnosis method for rollers based on a distributed fiber optic sensing system. The collected signals are processed and combined with an improved IForest algorithm to effectively detect the faulty rollers. Finally, on-site monitoring experiments were conducted on a 600 m long belt conveyor in a certain mine, and the monitoring results were obtained, indicating that the proposed method has a high recognition rate for faulty rollers and can effectively diagnose roller faults.

## A fault monitoring method for idler based on fiber optic sensors

Fiber optic itself is made of glass fiber, which has high resistance to most harsh chemical environments. Its surface is covered with a polymer or metal layer, which is one of its main advantages, especially in mining environments containing substances such as oil and gas. Compared with other sensors, optical fibers have more distinctive durability. In addition, fiber optic sensors are also known as distributed sensors, and the fibers in the optical cable serve as sensors that can measure temperature, strain, and sound waves^13^. Fiber optic sensors used for monitoring sound signals are commonly referred to as distributed acoustic sensors (DAS).

**Figure 2:**
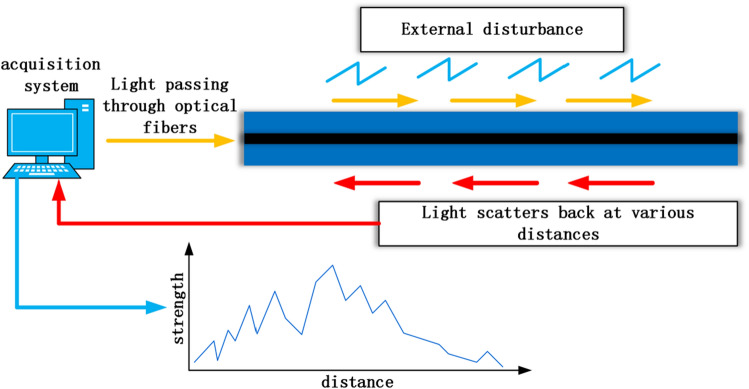
DAS principle of work.

The working principle of this sensor is the principle of backward scattering of light. Light is emitted from the light source and propagates through an optical fiber. When disturbance occurs at any distance along the optical fiber, the light will scatter back to the receiver. For acoustic monitoring, sound or vibration signals can be measured by backscattering light intensity (Rayleigh backscattering) similar to the frequency of emitted light^14^. The working principle of this system is based on the optical time-domain reflection method, where the position of the back scattered light is determined based on the time required for the receiver to receive the back scattered light after the light source is emitted. When the roller malfunctions, vibration occurs, and sound waves are transmitted to the tested optical cable, causing axial and radial strain on the fiber. The refractive index of the fiber changes correspondingly due to the influence of the elastic optical effect, and the phase of the Rayleigh scattering light in the fiber also changes, obtaining information on the phase changes of the Rayleigh scattering light in various parts of the fiber. Analyze the collected Rayleigh scattering light through the DAS system to determine the type of interference. Figure 2 illustrates the working principle of the DAS system.

Compared with references^9^,^10^,^11^, this technology can achieve real-time continuous monitoring of the rollers without the need for multiple sensors. The fiber itself acts as a continuous sensor and is passive.

## Signal processing analysis

### Fast Fourier Transform (FFT)

The application of Fast Fourier Transform (FFT) is very extensive, including digital signal processing, image processing, audio processing and other fields. It is one of the foundations of modern digital signal processing technology and also one of the core algorithms of many digital signal processing algorithms.

FFT is developed on the basis of Discrete Fourier Transform (DFT). Compared with DFT, FFT has a smaller computational complexity and is an efficient algorithm that avoids repetitive operations in DFT, greatly saving workload and enabling faster calculation of the signal spectrum.

The basic idea of FFT is to decompose DFT into smaller sub problems and recursively solve these sub problems. This recursive decomposition method greatly reduces the computational complexity of FFT, thereby improving computational efficiency.

After FFT, the discrete data signal results in the same number of complex points, and the physical meaning of each point changes from time and amplitude to frequency and amplitude. The amplitude and phase after Fourier transform^15^ are respectively represented as:

1 $$|F(w)| = \sqrt {R^{2} (w) + I^{2} (w)}$$

2 $$\varphi (w) = \arctan [I(w)/R(w)]$$

where: *R* (*w*) and *I* (*w*) are the real and imaginary parts of the Fourier transform | *F* (*w*) |.

Firstly, analyze the time-domain optical signals collected by the DAS system. Then, FFT is used to convert the time-domain signal into a frequency-domain signal.

### Short-time Fourier Transform

Short-time Fourier Transform (STFT) has been widely used in time–frequency analysis of time-varying and non-stationary signals, and is a collection of time-domain and frequency-domain characteristics.

Short time Fourier transform is the process of taking a certain length of time-domain signal as a window function, and further performing FFT on the intercepted time-domain signal to obtain the spectrogram over time period t. By sliding the window function over the detection time period, the collection of each spectral segment can be obtained. Therefore, Short time Fourier transform is a two-dimensional function of time and frequency^16^, and the basic calculation formula is as follows:

3 $$STFT_{{\text{f}}} \left( {t,f} \right) = \int_{ - \infty }^{\infty } {h\left( t \right)} p\left( {t - \beta } \right)e^{ - j\omega t} dt$$

where: *h* (*t*) is the time-domain signal, *P* (*t*- *β*) for the time window,with *β* centered, STFT is the multiplication of the vibration signal *h* (*t*) by a *β* FFT performed by the window function *p*(*t*-*β*) centered on the center,the area of the window function in the FFT is fixed. In order to improve the time-domain and frequency-domain resolution, the Hamming window function is selected for signal analysis in this paper.

### Isolation forest

The IForest algorithm is an ensemble learning algorithm based on decision trees, mainly used for anomaly detection and noise suppression. It identifies outliers in the data by dividing it into isolation point, while preserving important features of the data^17^. Compared with other anomaly detection methods such as statistics, clustering, nearest neighbors, etc., it neither calculates distance nor density, thus greatly reducing execution time and memory requirements, making this method suitable for large datasets and real-time processing.

**Figure 3:**
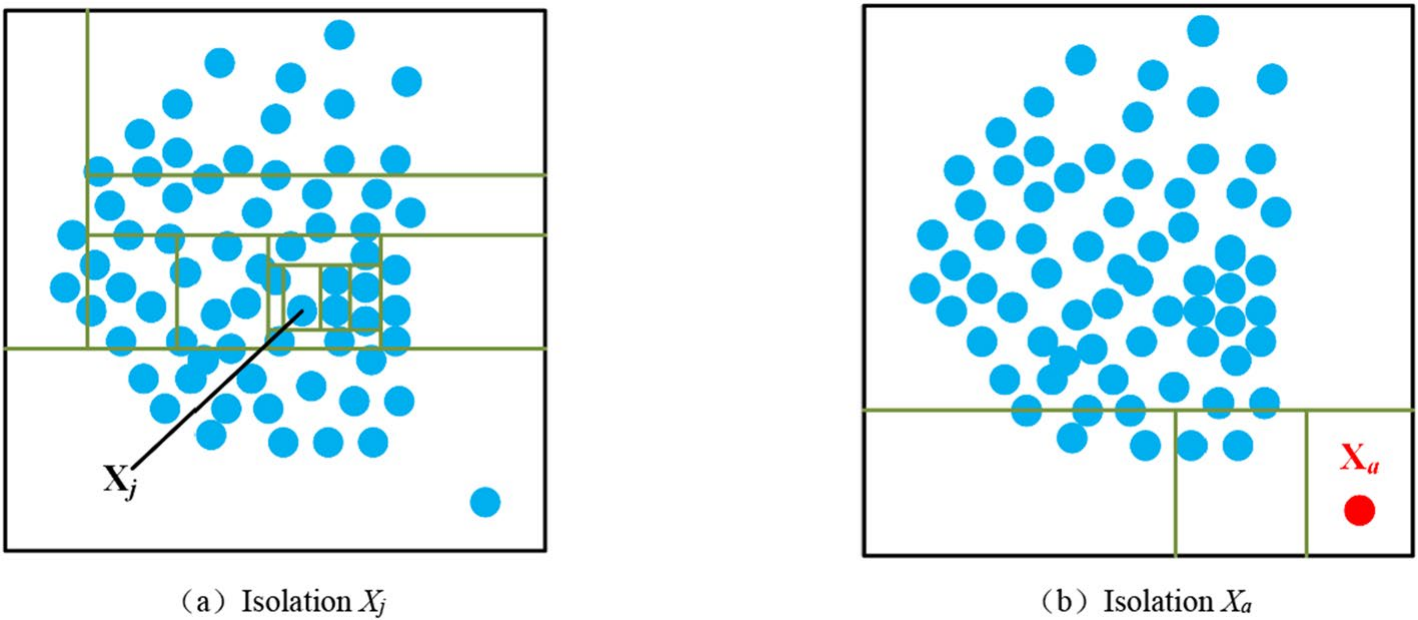
Normal data *X*_*j*_ and abnormal data *X*_*a*_ (**a**) Isolation *X*_*j*_ (**b**) Isolation *X*_*a*_*.*

Isolation is performed through continuous segmentation of the dataset. Figure 3 shows an example of abnormal data (*X*_*j*_) and normal data (*X*_*a*_). It can be seen that *X*_*j*_ is used more times to separate, while *X*_*a*_ is used less times to separate.

The forest framework is divided into a training phase and a testing phase. During the training phase, the dataset is sampled based on a predetermined subsampling size and recursively segmented until the instance is isolated (one branch only contains one data point) or reaches the Isolation Tree(ITree) height limit. The height limit of ITree can be estimated using the following expression^18^:

4 $${\text{limit}} = ceiling\left( {\log_{2} \psi } \right)$$

where: *ψ* for subsampling size.

According to literature^19^, using 28 or 256 subsampling sizes can provide sufficient anomaly detection details for a large range of data. Therefore, the neutron sampling size in this article is set to 256. The number of trees t determines the overall size, and the path length usually converges before t = 100. In this article, t = 100 is used as the default value.

During the testing phase, the average path length and anomaly score are used to determine anomalies. For each sample x, it is necessary to comprehensively calculate the results of each tree and calculate the anomaly score using the following formula:

5 $$s(x,y) = 2^{{ - \frac{E(h(x))}{{c(y)}}}}$$

where in:

6 $$c(y) = 2h(y - 1) - (2(y - 1)/y)$$

7 $$c(y) = H(k) = e + \ln (k)$$

where: *s* (*x*, *y*) is the anomaly score of *x* obtained by ITree from the training data of y samples, with a range of [0,1], *H* (*x*) is the height of *x* on each tree, *E* (*h* (*x*)) is the average path length of *x*, *c* (*y*) is the average path length of a given number of samples *y*, obtained from the average search length of failed searches in a binary search tree^20^, used to standardize *h* (*x*), *H* (*k*) is the harmonic value, e is the Euler constant, e = 0.5772156649.

**Figure 4:**
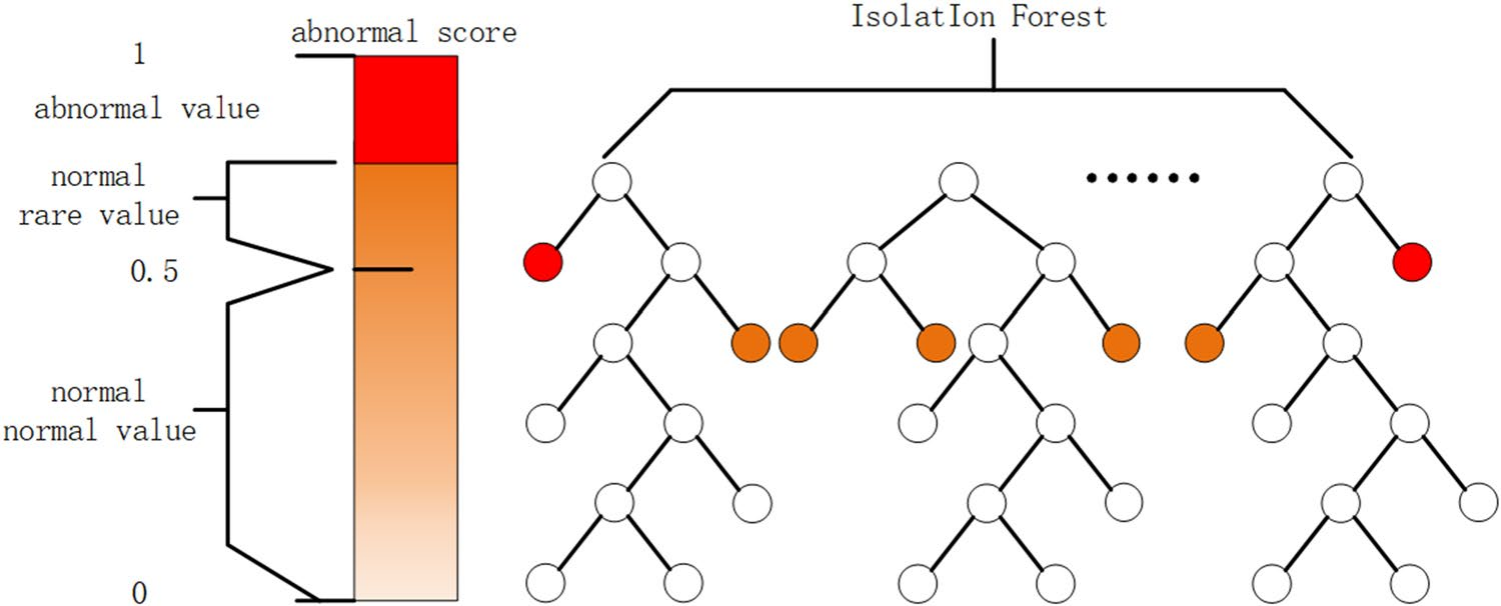
Schematic diagram of abnormal score allocation.

According to Eq. (5), in general, if* E* (*h* (*x*)) approaches *c* (*y*) and s approaches 0.5, then the sample *x* may not contain any identifiable outliers; If *E* (*h* (*x*)) approaches 0 and *s* approaches 1, then data *x* is identified as abnormal data; If *E* (*h* (*x*)) approaches *y*-1 and *s* approaches 0, then data *x* is recognized as normal data. The schematic diagram of abnormal score allocation is shown in Fig. 4. In practice, abnormal decisions are made when *s* is greater than 0.5, which may lead to false positives because the optimal decision threshold is not always equal to 0.5. In order to ensure the accuracy of the model, this article will further study the threshold values.

In addition, this algorithm can be applied to various application scenarios, such as network security monitoring^21^, diesel engine fault monitoring^17^, motor fault monitoring^22^, and so on.

### Improved isolation forest

**Figure 5:**
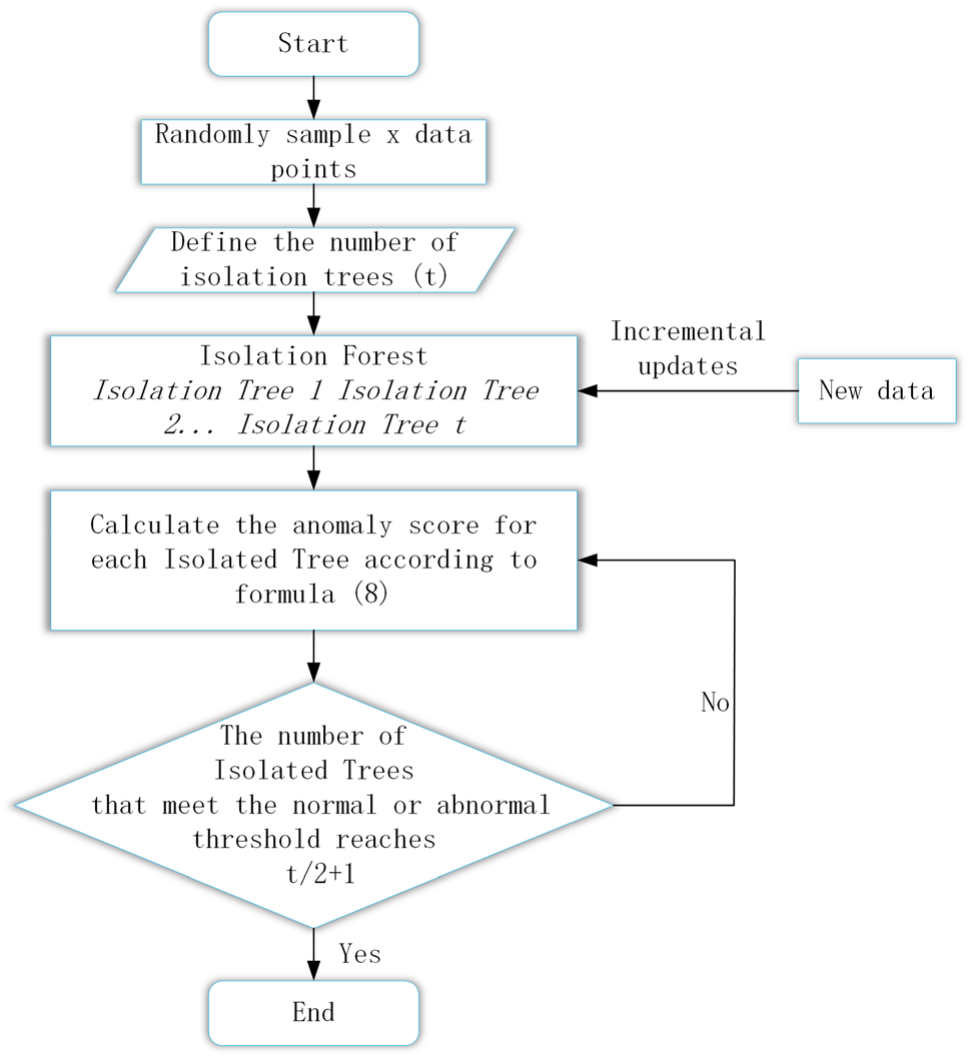
Improved IForest framework diagram.

This article improves the traditional IForest algorithm and proposes an IMV-IForest algorithm. The improved forest framework is shown in Fig. 5.

Specific ideas:

1. Forest construction stage: Use traditional IForest algorithms to construct some effective ITree on the initial dataset as the initial model. When new data points arrive, only update the existing ITree for the newly added data and add a new ITree^23^.
2. During the scoring stage, calculate the score *x* for each tree *j*. This process is repeated *x* times in each tree in the forest until a majority decision is made, meaning that the majority of trees corresponds to *t*/2 + 1 trees, and the final decision is made.
  8 $$s_{j} (x,y) = 2^{{ - \frac{h(x)}{{c(y)}}}}$$

Compared with traditional IForest, this method avoids the need to repeatedly construct the entire forest and does not require calculating the paths of all trees in the forest to obtain scores. Instead, it calculates the score x for each tree j and compares it with the threshold score. When most trees classify data items as abnormal or normal, the final decision can be made, effectively reducing execution time and memory.

## On site data collection and analysis

### Introduction to the experimental site

Two months of data collection work has been carried out on the belt conveyor of a mine. The main parameters of the conveyor are shown in Table 1.

**Table 1:** Main technical parameters of conveyor.

Serial number	Project	Unit	Vertical
One	Bandwidth	mm	1600
Two	Volume	t/h	2400
Three	Belt speed	m/s	4
Four	Angle	°	0 ~ 14
Five	Length	m	600

The experimental conveyor is a groove conveyor, mainly equipped with groove bearing rollers, V-shaped lower rollers, upper center rollers, V-shaped lower center rollers, etc. The spacing between the bearing rollers is about 1500 mm, and the spacing between the V-shaped lower rollers is about 3000 mm.

**Figure 6:**
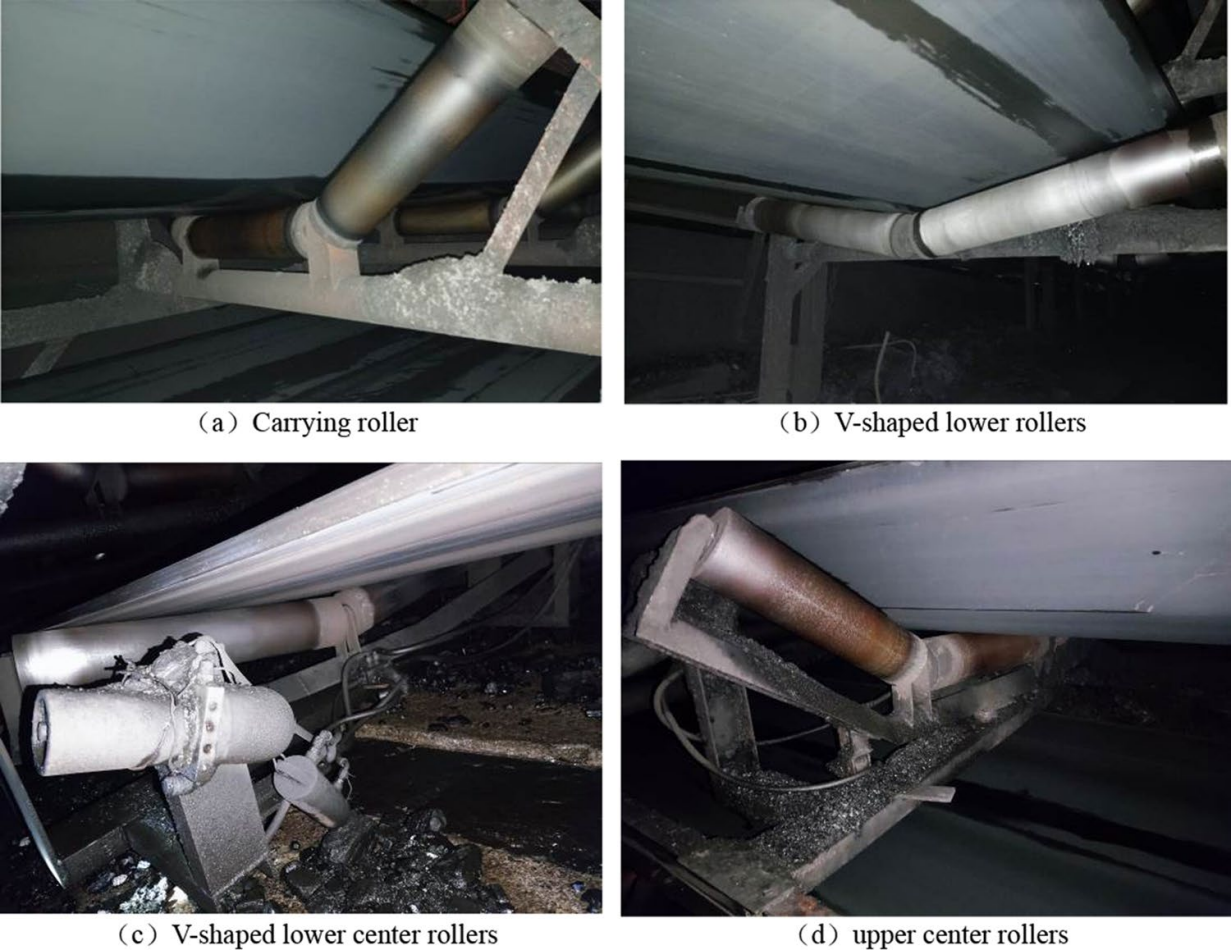
Roller type. (**a**) Carrying roller, (**b**) V-shaped lower rollers, (**c**) V-shaped lower center rollers, (**d**) upper center rollers.

The supporting rollers of the belt conveyor are shown in Fig. 6.

At present, the mining company arranges transportation team personnel to patrol along the conveyor every day, detecting roller faults through listening to sound and visual inspection, in order to timely detect faulty rollers.

### Experimental site layout

**Figure 7:**
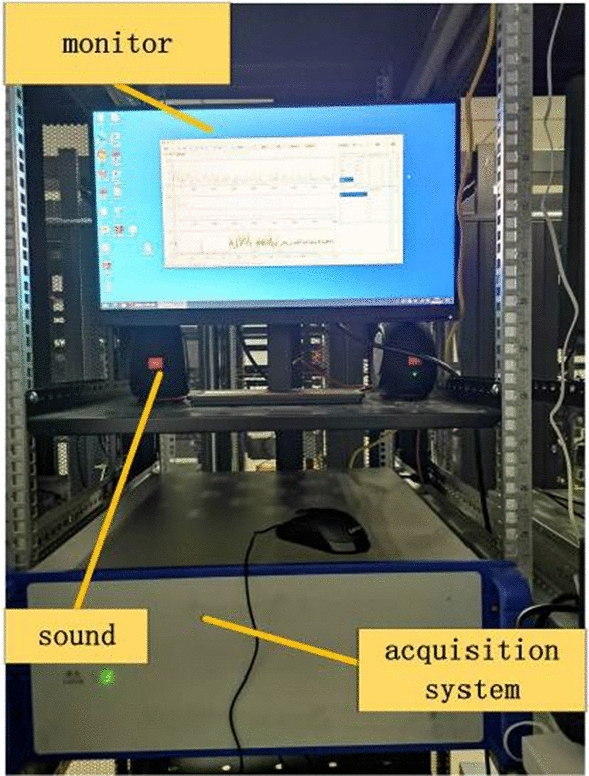
DAS acquisition system.

This study used a DAS acquisition system as shown in Fig. 7, which has a spatial resolution of 4 m, a transmission distance of about 10 km, and a sampling rate set at 16 kHz.

**Figure 8:**
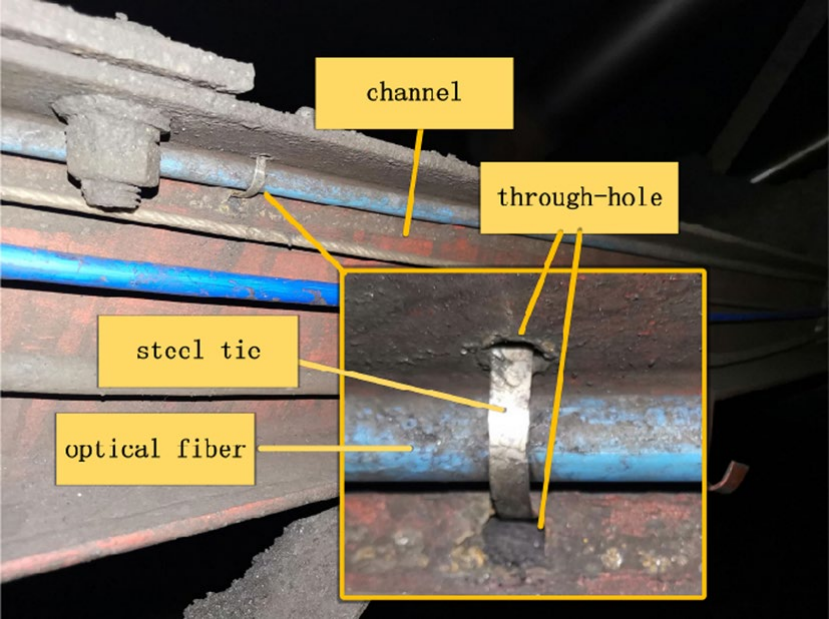
Fiber optic installation position and fixing method.

Due to coal falling on the test site, optical fibers were installed on the upper side of the channel steel on both sides of the conveyor frame in this experiment. 8 mm through-holes were drilled on the inner side of the upper side of the frame every 800 mm for fixing the optical fibers with zip ties, which can prevent them from being smashed, as shown in Fig. 8.

After the installation of the collection system is completed, on-site calibration is carried out to determine the position of each frame of the conveyor in the system, with the first and last frame numbers being 9 and 184, respectively. To ensure the accuracy of positioning, calibration tests are conducted every 10 racks on site, and random calibration tests are conducted between every 10 racks.

### Analysis of manual inspection records

According to the manual inspection records in Table 2, the main faults on site were the detachment of the roller bearings and the breakage of the roller cylinder skin, with the most common being the detachment of the roller bearings. The following will mainly focus on specific analysis of these two types of faults.

**Table 2:** Manual inspection records.

Inspection time	Number of inspection racks	Number of manual inspection failures	Fault type
First month	5456	2	Roller skin malfunction
		35	Roller bearing failure
Second month	5456	5	Roller skin malfunction
		28	Roller bearing failure

Extract data from the data collected over the past 2 months for 1 week before and after the malfunction of shelves 58 and 24 for specific analysis.

#### Roller bearing failure

**Figure 9:**
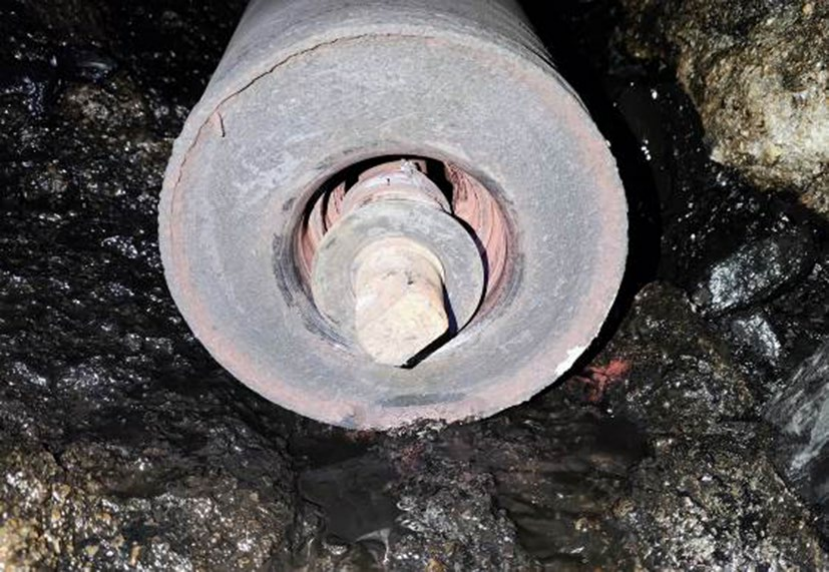
Malfunction roller of rack 58.

During manual inspection, it can be heard that the sound of the rack here is different from that of a normal rack. Pedestrian side shelf 58 reported a bearing detachment fault, as shown in Fig. 9 for the faulty roller.

**Figure 10:**
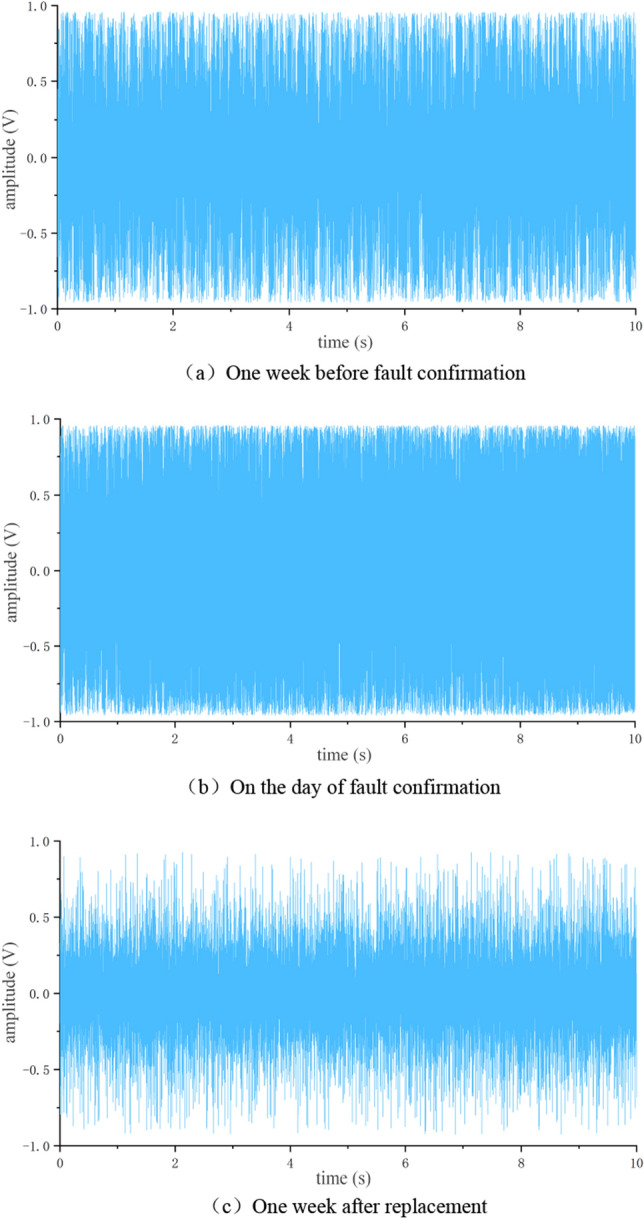
Time domain signal of rack 58. (**a**) 1 week before fault confirmation, (**b**) On the day of fault confirmation, (**c**) 1 week after replacement.

Further analyze the collected fault data and normal data of shelf 58. Firstly, normalize the data and generate a time-domain graph, as shown in Fig. 10.

From Fig. 10a, b, it can be seen that when the supporting roller malfunctions, the amplitude changes dramatically within [− 1,1], and there is an oversaturation situation. The vibration amplitude in Fig. 10c is smaller than the variation in (a, b), but there is still no obvious distinguishing feature, which needs further analysis.

**Figure 11:**
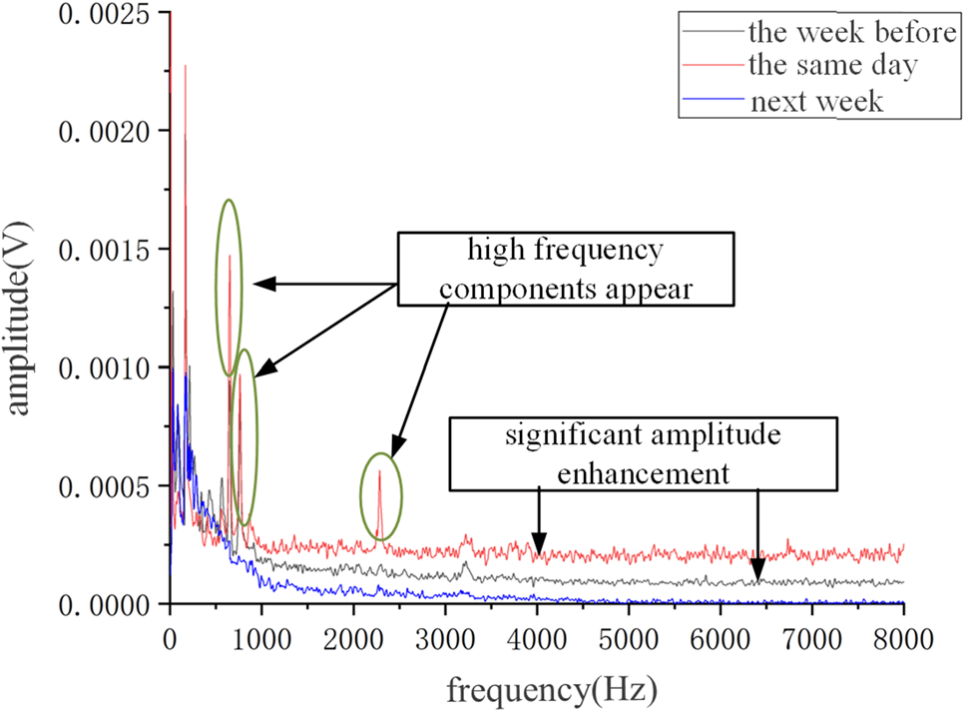
Frequency domain signal of fault on shelf 58.

Perform FFT transformation on it to obtain its frequency domain diagram, as shown in Fig. 11. It can be seen that there is no variation pattern in the vibration amplitude at three different times at low frequencies. At high frequencies, after the replacement of the faulty roller, the overall amplitude of the frequency domain diagram of frame 58 was small. Before the confirmation of the roller fault, the overall amplitude increased, and a small amount of high-frequency components appeared. On the day of fault confirmation, it was found that the overall amplitude significantly increased, and the high-frequency components increased. The high-frequency components were mainly concentrated in 500–2500 Hz.

**Figure 12:**
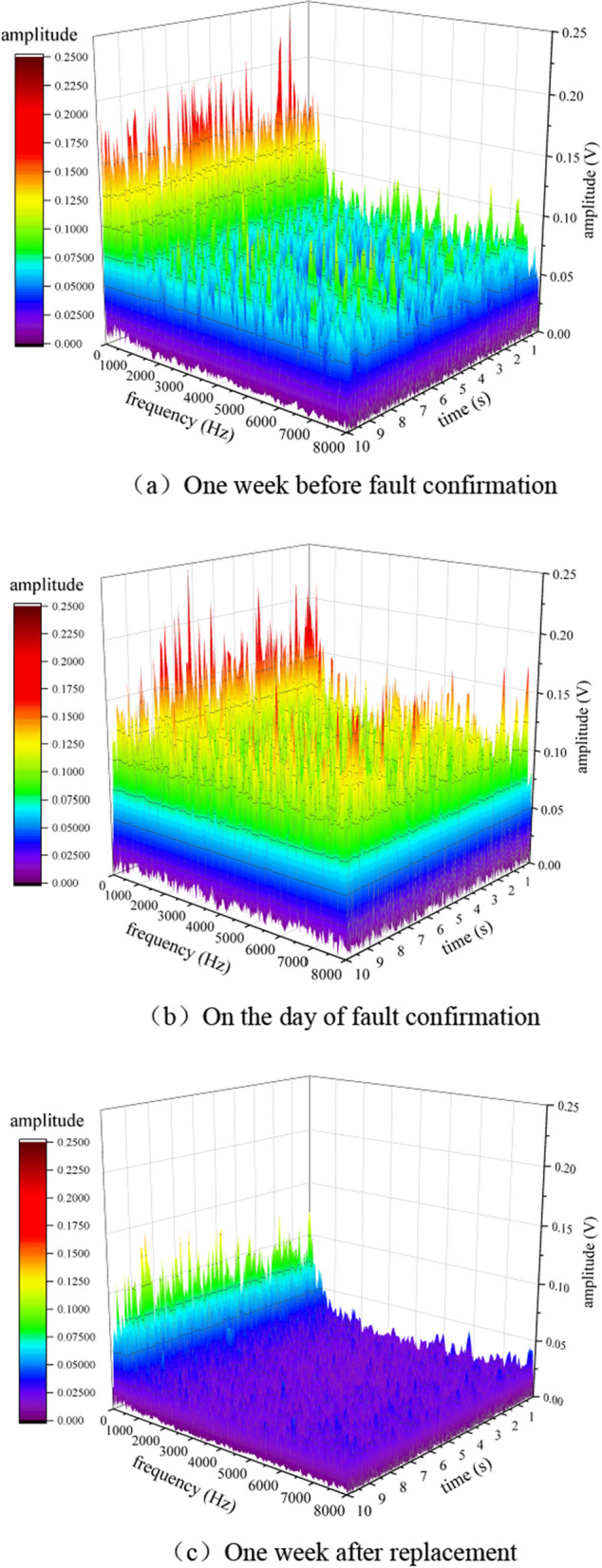
Time frequency domain signal of shelf 58**.** (**a**) 1 week before fault confirmation, (**b**) On the day of fault confirmation, (**c**) 1 week after replacement.

Due to the fact that FFT belongs to static analysis, it is impossible to determine the occurrence time of a certain high-frequency component. In order to obtain the dynamic information of the signal, a short-time Fourier transform is used to perform time–frequency domain analysis on the above three signals, as shown in Fig. 12. From the graph, it can be seen that before the fault was confirmed, the frequency of the vibration signal significantly increased compared to the normal frequency spectrum of the supporting roller at various times. On the day of fault confirmation, the difference in low-frequency energy was not significant compared to before confirmation, while the difference in high-frequency energy was significant. On the day of fault confirmation, the vibration signal frequency did not differ significantly at different times.

#### Roller skin malfunction

The main reason for the failure of the roller skin is due to the high friction resistance between the roller and the conveyor belt, and the wear of the edge or middle contact area, which leads to the thinning of the contact area skin and causes the roller skin to break. Secondly, the operating environment of the rollers is harsh, and coal falls seriously on site. When the coal at the bottom of the conveyor reaches a certain height, it will directly contact and friction with the rollers, causing damage to the roller skin. This type of malfunction is detected through auditory and visual inspection during inspections.

**Figure 13:**
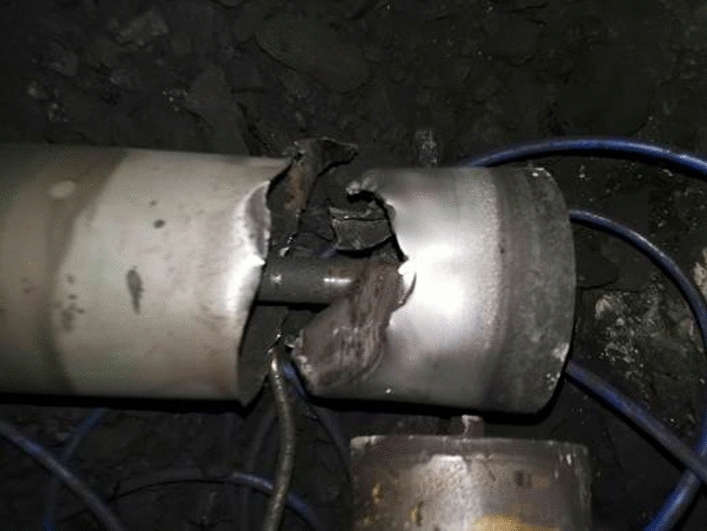
Malfunction roller of rack 24.

The non pedestrian side shelf No. 24 has been confirmed to have a broken cylinder skin fault, and the faulty roller is shown in Fig. 13.

**Figure 14:**
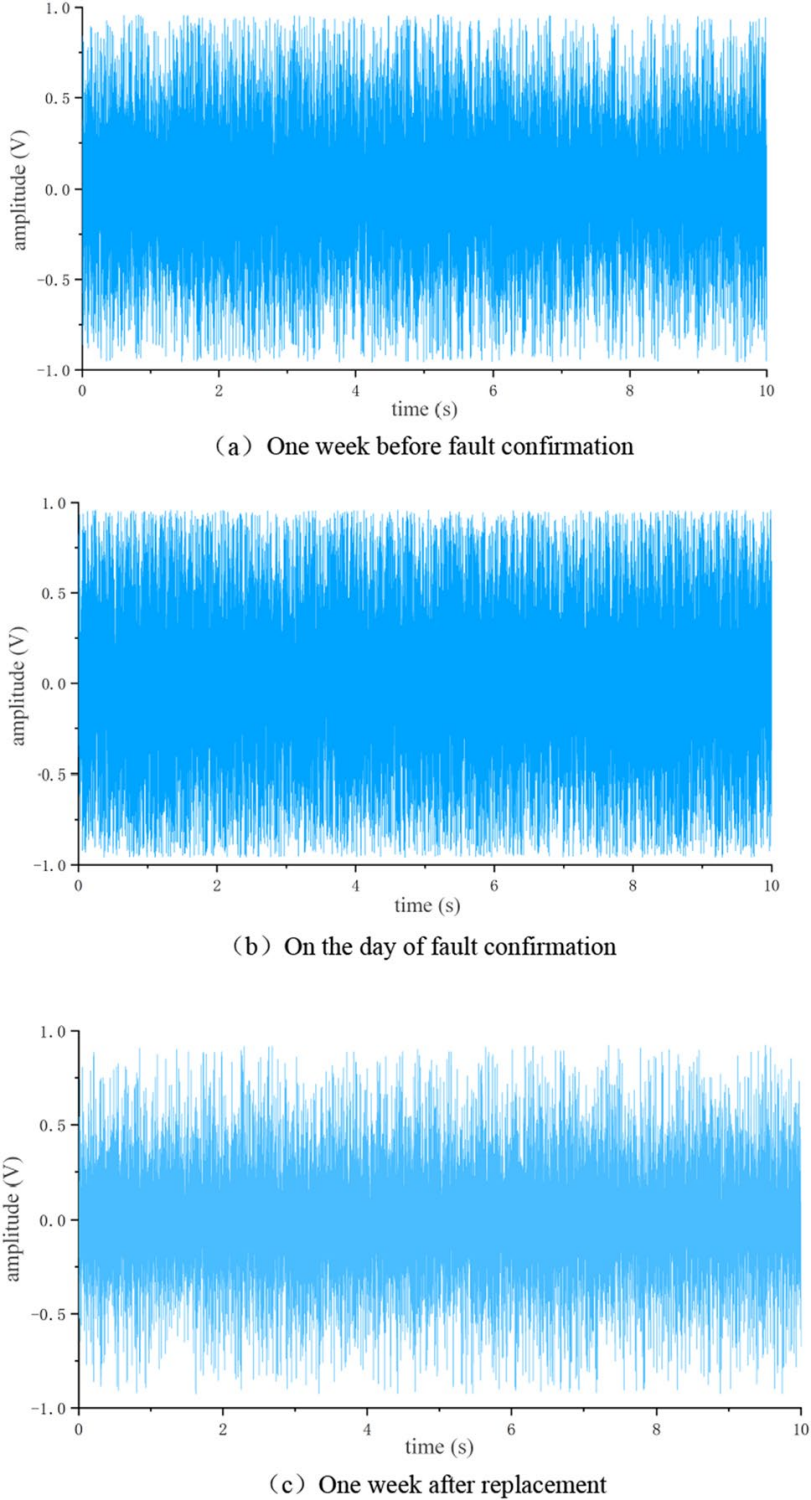
Time domain signal of rack 24**.** (**a**) One week before fault confirmation, (**b**) On the day of fault confirmation, (**c**) One week after replacement.

Using the same processing method as in section "Roller bearing failure", generate a time-domain graph as shown in Fig. 14.

From Fig. 14, it can be seen that the vibration amplitude before and on the day of fault confirmation was smaller than the vibration amplitude of bearing detachment. After replacing the faulty roller, the vibration amplitude was not significantly different from that of frame 58.

**Figure 15:**
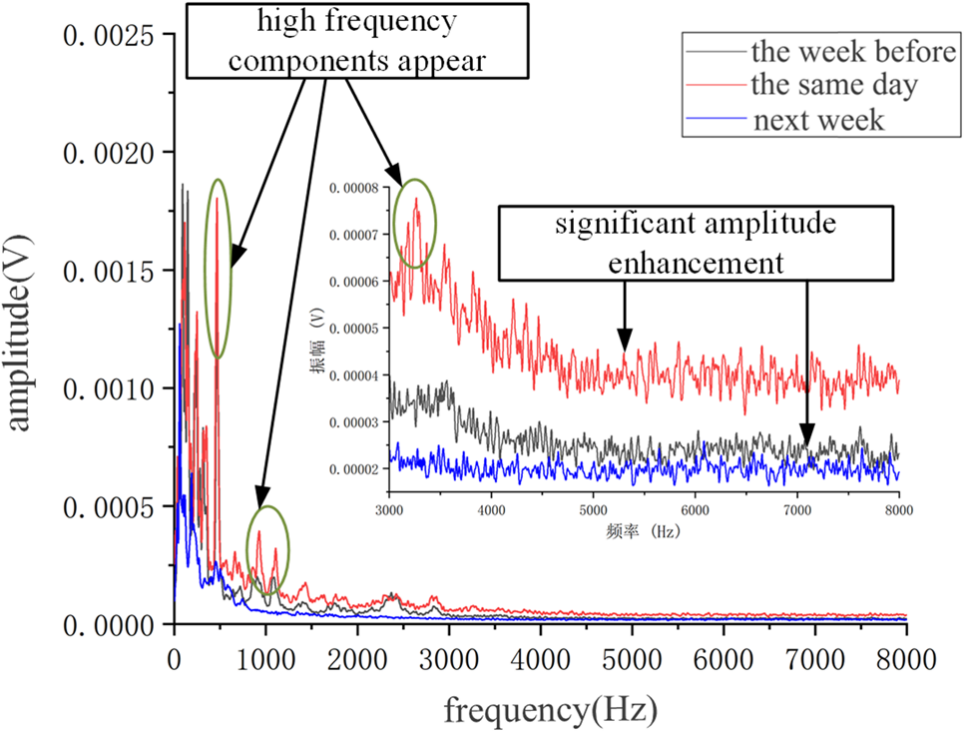
Frequency domain signal of fault on shelf 24.

Perform FFT transformation on it to obtain its frequency domain diagram, as shown in Fig. 15. It can be seen that there is no obvious change pattern in the low frequency under these three types of times. The overall amplitude of the frequency domain diagram of the replaced roller at the high frequency is small, while the overall amplitude before the roller fault confirmation increases. On the day of fault confirmation, it is found that the overall amplitude significantly increases, and the high frequency component increases, mainly concentrated in 500–3000 Hz.

**Figure 16:**
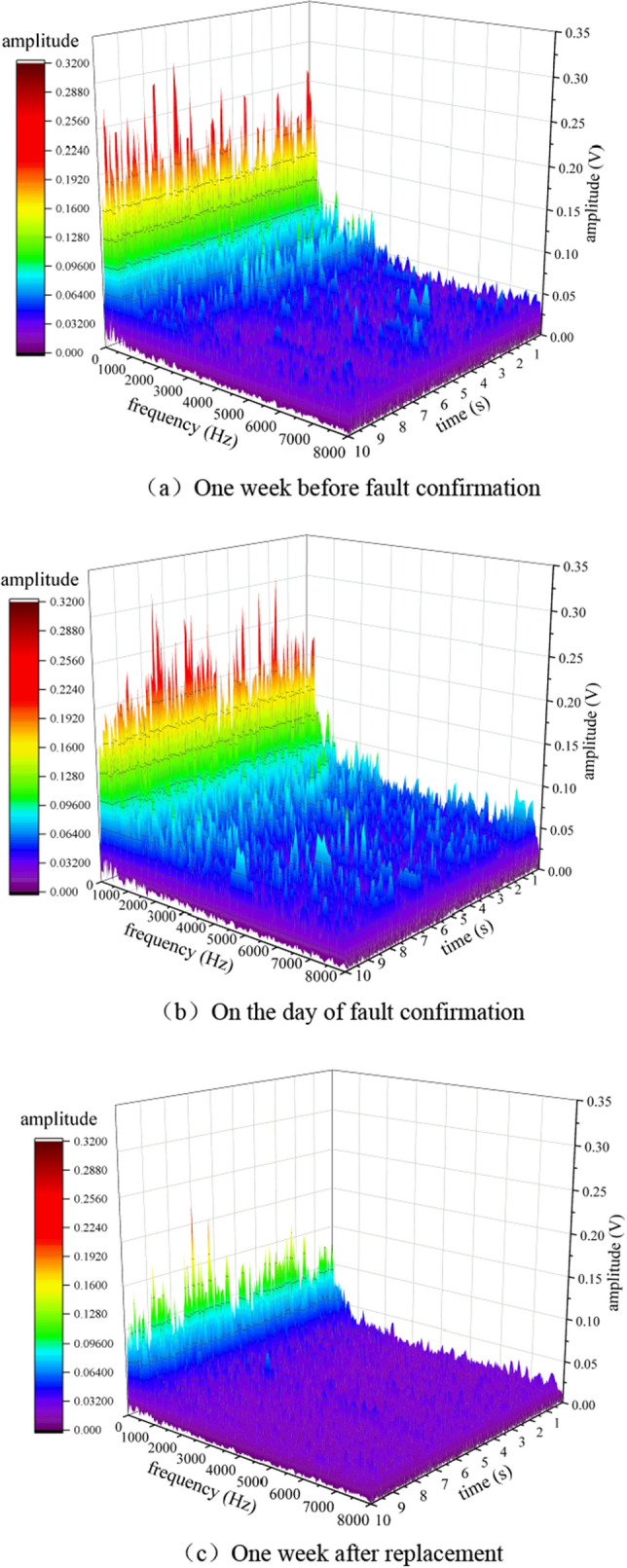
Time frequency domain signal of shelf 24. (**a**) One week before fault confirmation, (**b**) On the day of fault confirmation, (**c**) 1 week after replacement.

Using short-time Fourier transform to perform time–frequency domain analysis on the above three signals, as shown in Fig. 16. From the graph, it can be seen that before the fault was confirmed, the frequency of the vibration signal significantly increased compared to the normal frequency spectrum of the supporting roller at various times. On the day of fault confirmation, the difference in low-frequency energy was not significant compared to before, while the difference in high-frequency energy was significant. Compared with the failure of the roller bearing detachment, the vibration energy of the roller fracture fault at high frequencies is significantly smaller than that of the roller bearing detachment fault.

### Abnormal score analysis

**Figure 17:**
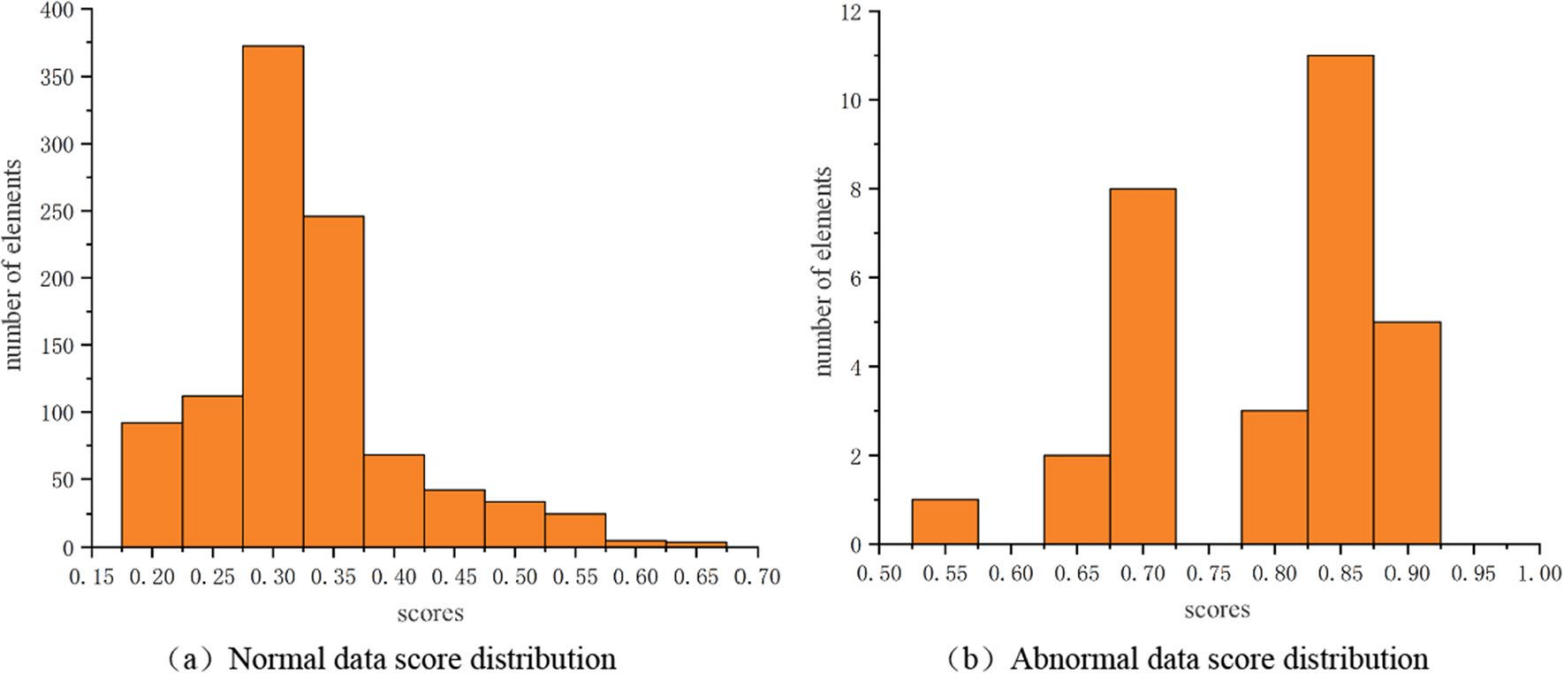
Distribution of data scores. (**a**) Normal data score distribution (**b**) Abnormal data score distribution.

In practice, abnormal score analysis involves making decisions when the score exceeds a certain threshold, typically 0.5. However, this rule can lead to false positives or false negatives, as the optimal decision threshold is not always equal to 0.5. To determine the optimal alarm threshold, we selected 5000 normal samples and 70 faulty samples from the collected data to form a dataset. Among these, 30 faulty samples and 1000 normal samples were used to analyze the distribution of anomaly scores, as depicted in Fig. 17.

The abnormal score distribution of these data is shown in Fig. 17, where (a) is the normal data score distribution, and (b) is the abnormal data score distribution. The normal data scores are concentrated between 0.175 and 0.675, and the abnormal data scores are concentrated between 0.525 and 0.925. Therefore, a threshold was selected between 0.525 and 0.675, and after repeated experiments, the threshold was ultimately set to 0.60.

## Algorithm comparison

### Evaluating indicator

ROC (Receiver Operating Characteristic) characteristic curve refers to the line connecting multiple points obtained under specific conditions, with the False Positive Rate (FPR) obtained under different judgment criteria as the x-axis and the True Positive Rate (TPR) as the y-axis. AUC is a single number that summarizes the overall performance of a classifier, with higher values indicating better performance.

True positive (*T*_*p*_), false negative (*F*_*n*_), true negative (*T*_*n*_), false positive (*F*_*p*_), so precision (*P*) and recall (*R*) can be defined^24^:

9 $$P = \frac{{T_{p} }}{{T_{p} + F_{p} }}$$

10 $$R = \frac{{T_{p} }}{{T_{p} + F_{n} }}$$

The higher the accuracy, the fewer false alarms; The higher the recall rate, the fewer missed alarms, which is a contradictory measure. In order to comprehensively consider these two indicators, the *F*_1_ indicator^17^ is introduced:

11 $$F_{1} = 2 \times \frac{PR}{{P + R}}$$

### Evaluate results

The above datasets were used for comparative experiments in the traditional IForest, E-IForest, and improved IForest framework, and the results are shown in Table 3.

**Table 3:** Comparison results.

Algorithm	IForest	E-IForest	IMV-IForest
Time(s)	8.32	2.45	1.02
*F*_1_ score	0.81	0.85	0.98

Table 3 shows the actual running time and *F*_1_ scores of the three methods. It can be seen that the IMV-IForest algorithm proposed in this paper has significant advantages in computational time and algorithm accuracy compared to the other two algorithms.

## On site test verification

In order to verify the effectiveness of the proposed scheme, a three-month monitoring experiment was conducted.

**Figure 18:**
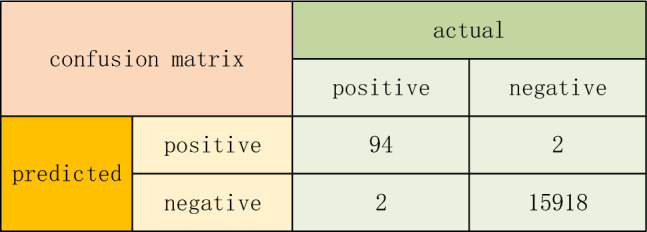
Confusion matrix.

**Figure 19:**
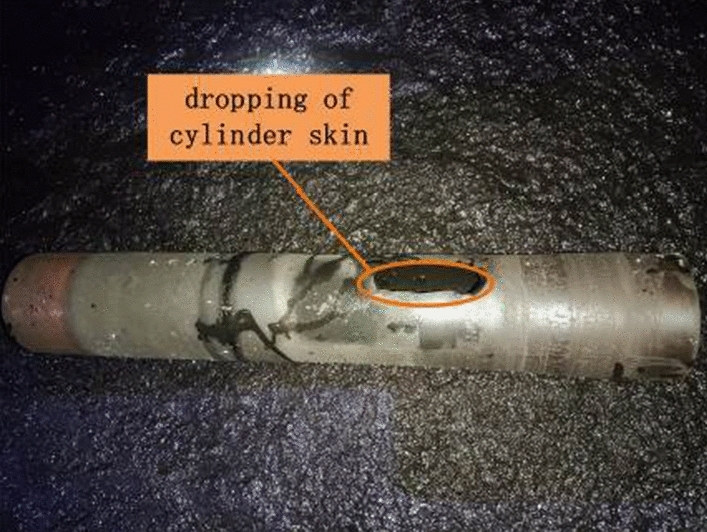
Dropping roller skin.

**Figure 20:**
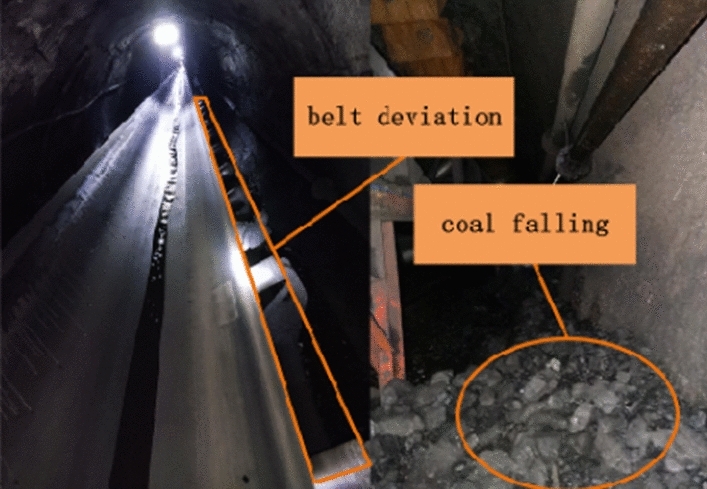
Belt deviation and coal falling.

**Figure 21:**
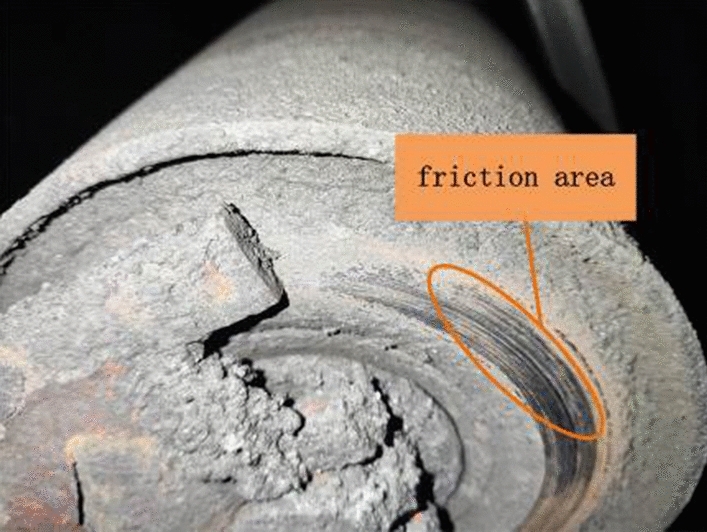
Roller friction frame.

The on-site testing results are shown in Table 4. Through statistical analysis of the on-site testing situation, the confusion matrix for evaluating the effectiveness is obtained, as shown in Fig. 18. As shown in Fig. 18, the accuracy rate of detecting faulty idlers during these three months is 97.92%. The actual failure that was not reported was due to a small part of the cylinder skin falling off, which was discovered through manual visual inspection. There was no obvious vibration or abnormal sound during on-site operation, as shown in Fig. 19. The actual fault reported was due to the deviation of the on-site belt, which caused severe coal accumulation in the local area, as shown in Fig. 20. At the same time, there was also a phenomenon of scratches between the side of the roller and the roller frame, as shown in Fig. 21.

**Table 4:** On site inspection results.

Inspection time	Number of inspection racks	Number of manual inspection failures	The method in this article accurately reports the number of faults and the number of false alarms	Number of faults not reported
First month	5280	37	36	1
Second month	5456	33	33	1
Third month	5280	26	25	0

## Economic benefits

This method can monitor the belt conveyor system in real-time, and after application, it can detect faulty rollers about a week in advance. At the same time, it can reduce or even eliminate economic losses and production accidents caused by delayed detection of roller faults. It can effectively reduce the intensity of manual inspections, reduce unplanned shutdowns of belt conveyors, and reduce belt wear and loss. Moreover, the monitoring system is passive on site and does not introduce additional risk sources, Suitable for the needs of coal mining application scenarios, it is of great significance for promoting the construction of intelligent, safe, and efficient mining production systems.

## Conclusion

This article proposes a fault diagnosis method for rollers based on a distributed fiber optic sensing system. Firstly, the collected signal is processed and combined with an improved IForest algorithm. At the same time, it is compared with other IForest algorithms to effectively detect faulty rollers. Finally, on-site monitoring experiments were conducted on a 600 m long belt conveyor in a certain mine, and the monitoring results were obtained. The specific conclusions are as follows:

1. A framework based on IMV-IForest is proposed, which has higher efficiency and accuracy compared to traditional IForest and E-IForest.
2. The variation pattern of roller data over time and space can be used for early prediction of roller faults.
3. By comparing the results of on-site experiments with manual testing, it has been proven that the proposed method has a high accuracy, with a detection accuracy of 97.92%, and can effectively detect roller faults.

In the future, we plan to conduct more in-depth research on fiber optic micro vibration monitoring, in order to better monitor the rollers of belt conveyors and contribute to the coal mining industry.

## Data Availability

All data generated or analyzed in this study are included in this article. For more information, please contact the corresponding author.
